# Association of accompanying dyspnoea with diagnosis and outcome of patients presenting with acute chest discomfort

**DOI:** 10.1093/ehjacc/zuad026

**Published:** 2023-03-14

**Authors:** Jasper Boeddinghaus, Thomas Nestelberger, Luca Koechlin, Pedro Lopez-Ayala, Desiree Wussler, Maximilian Mais, Luca Zwimpfer, Tobias Zimmermann, Karin Wildi, Maria Rubini Giménez, Ivo Strebel, Òscar Miró, F Javier Martin-Sanchez, Jiri Parenica, Dagmar I Keller, Danielle M Gualandro, Christian H Nickel, Roland Bingisser, Michael Christ, Christian Mueller, for the APACE investigators

**Affiliations:** Cardiovascular Research Institute Basel (CRIB) and Department of Cardiology, University Hospital Basel, University of Basel, Petersgraben 4, CH-4031 Basel, Switzerland; GREAT network, Rome, Italy; BHF Centre for Cardiovascular Science, University of Edinburgh, Scotland, United Kingdom; Cardiovascular Research Institute Basel (CRIB) and Department of Cardiology, University Hospital Basel, University of Basel, Petersgraben 4, CH-4031 Basel, Switzerland; GREAT network, Rome, Italy; Cardiovascular Research Institute Basel (CRIB) and Department of Cardiology, University Hospital Basel, University of Basel, Petersgraben 4, CH-4031 Basel, Switzerland; GREAT network, Rome, Italy; Department of Cardiac Surgery, University Hospital Basel, University of Basel, Switzerland; Cardiovascular Research Institute Basel (CRIB) and Department of Cardiology, University Hospital Basel, University of Basel, Petersgraben 4, CH-4031 Basel, Switzerland; GREAT network, Rome, Italy; Cardiovascular Research Institute Basel (CRIB) and Department of Cardiology, University Hospital Basel, University of Basel, Petersgraben 4, CH-4031 Basel, Switzerland; GREAT network, Rome, Italy; Division of Internal Medicine, University Hospital Basel, University of Basel, Switzerland; Cardiovascular Research Institute Basel (CRIB) and Department of Cardiology, University Hospital Basel, University of Basel, Petersgraben 4, CH-4031 Basel, Switzerland; Cardiovascular Research Institute Basel (CRIB) and Department of Cardiology, University Hospital Basel, University of Basel, Petersgraben 4, CH-4031 Basel, Switzerland; Cardiovascular Research Institute Basel (CRIB) and Department of Cardiology, University Hospital Basel, University of Basel, Petersgraben 4, CH-4031 Basel, Switzerland; GREAT network, Rome, Italy; Department of Intensive Care Medicine, University Hospital Basel, University of Basel, Basel, Switzerland; Cardiovascular Research Institute Basel (CRIB) and Department of Cardiology, University Hospital Basel, University of Basel, Petersgraben 4, CH-4031 Basel, Switzerland; GREAT network, Rome, Italy; Critical Care Research Group, The Prince Charles Hospital, Brisbane and the University of Queensland, Brisbane, Australia; Cardiovascular Research Institute Basel (CRIB) and Department of Cardiology, University Hospital Basel, University of Basel, Petersgraben 4, CH-4031 Basel, Switzerland; GREAT network, Rome, Italy; Leipzig Heart Center, Leipzig, Germany; Cardiovascular Research Institute Basel (CRIB) and Department of Cardiology, University Hospital Basel, University of Basel, Petersgraben 4, CH-4031 Basel, Switzerland; GREAT network, Rome, Italy; GREAT network, Rome, Italy; Emergency Department, Hospital Clinic, Barcelona, Catalonia, Spain; GREAT network, Rome, Italy; Servicio de Urgencias, Hospital Clínico San Carlos, Madrid, Spain; Department of Cardiology, University Hospital Brno, Brno, Czech Republic and Medical Faculty, Masaryk University, Brno, Czech Republic; Emergency Department, University Hospital Zurich, Zurich, Switzerland; Cardiovascular Research Institute Basel (CRIB) and Department of Cardiology, University Hospital Basel, University of Basel, Petersgraben 4, CH-4031 Basel, Switzerland; GREAT network, Rome, Italy; Emergency Department, University Hospital Basel, University of Basel, Switzerland; Emergency Department, University Hospital Basel, University of Basel, Switzerland; Emergency Department, Kantonsspital Luzern, Switzerland; Cardiovascular Research Institute Basel (CRIB) and Department of Cardiology, University Hospital Basel, University of Basel, Petersgraben 4, CH-4031 Basel, Switzerland; GREAT network, Rome, Italy

**Keywords:** Dyspnoea, High-sensitivity cardiac troponin, 0/1h-algorithm, Diagnosis of MI

## Abstract

**Aims:**

The presence of accompanying dyspnoea is routinely assessed and common in patients presenting with acute chest pain/discomfort to the emergency department (ED). We aimed to assess the association of accompanying dyspnoea with differential diagnoses, diagnostic work-up, and outcome.

**Methods and results:**

We enrolled patients presenting to the ED with chest pain/discomfort. Final diagnoses were adjudicated by independent cardiologists using all information including cardiac imaging. The primary diagnostic endpoint was the final diagnosis. The secondary diagnostic endpoint was the performance of high-sensitivity cardiac troponin (hs-cTn) and the European Society of Cardiology (ESC) 0/1h-algorithms for the diagnosis of myocardial infarction (MI). The prognostic endpoints were cardiovascular and all-cause mortality at two years. Among 6045 patients, 2892/6045 (48%) had accompanying dyspnoea. The prevalence of acute coronary syndrome (ACS) in patients with vs. without dyspnoea was comparable (MI 22.4% vs. 21.9%, *P* = 0.60, unstable angina 8.7% vs. 7.9%, *P* = 0.29). In contrast, patients with dyspnoea more often had cardiac, non-coronary disease (15.3% vs. 10.2%, *P* < 0.001). Diagnostic accuracy of hs-cTnT/I concentrations was not affected by the presence of dyspnoea (area under the curve 0.89–0.91 in both groups), and the safety of the ESC 0/1h-algorithms was maintained with negative predictive values >99.4%. Accompanying dyspnoea was an independent predictor for cardiovascular and all-cause death at two years [hazard ratio 1.813 (95% confidence intervals, 1.453–2.261, *P* < 0.01)].

**Conclusion:**

Accompanying dyspnoea was not associated with a higher prevalence of ACS but with cardiac, non-coronary disease. While the safety of the diagnostic work-up was not affected, accompanying dyspnoea was an independent predictor for cardiovascular and all-cause death.

**Clinical Trial Registration:**

https://clinicaltrials.gov/ct2/show/NCT00470587, number NCT00470587

## Graphical Abstract

**Graphical abstract:**
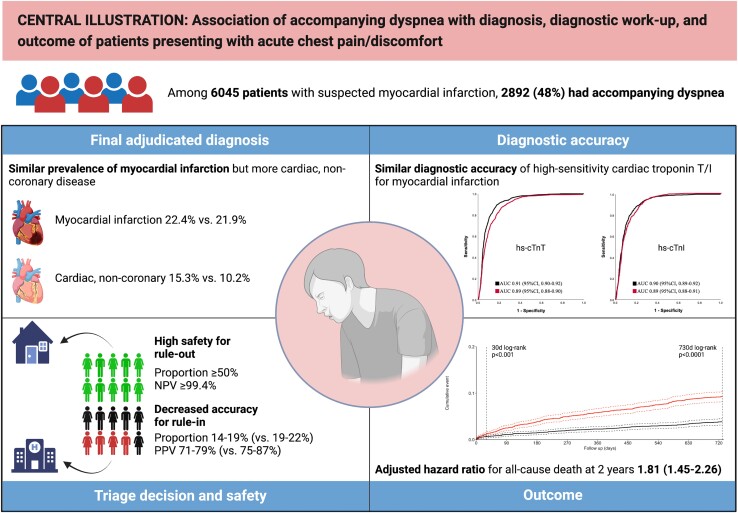

**In line with the Journal’s conflict of interest policy, this paper was handled by Borja Ibanez.**

## Introduction

Acute myocardial infarction (AMI) remains the leading cause of premature death worldwide.^1–3^ Each year, about 15 million patients present to the emergency department (ED) with acute chest pain/discomfort, the cardinal symptom of AMI.^1–3^ Rapid identification of AMI as a life-threatening cause of acute chest pain/discomfort is crucial for the early initiation of highly effective evidence-based therapy. Electrocardiography (ECG) and cardiac troponin (cTn) form the diagnostic cornerstones and complement clinical assessment in the ED.^1,3–11^

A relevant proportion of patients presenting to the ED with acute chest pain/discomfort do also complain about dyspnoea. Unfortunately, the impact of accompanying dyspnoea on the prevalence of AMI, the differential diagnosis, the diagnostic accuracy of hs-cTnT/I concentrations, and the hs-cTnT/I-based rapid algorithms, as well as the short- and long-term outcomes of these patients is largely unknown. Pilot studies, most of which had limited granularity in patient phenotyping and exclusively investigated dyspnoea as an alternative to chest pain/discomfort as the presenting symptom, but did not evaluated dyspnoea as an accompanying symptom, suggested that the presence of dyspnoea may impact on the final diagnosis and may be associated with worse outcome.^12–18^

To address this major gap in knowledge, we addressed these research questions within a large international multicentre diagnostic study that prospectively assessed the presence of accompanying dyspnoea in patients presenting with acute chest pain/discomfort to the ED.

## Methods

### Study design and population

This was a secondary analysis from a prospective international multicentre study including 12 centres in five countries aiming at advancing the early diagnosis of AMI (NCT00470587).^19–23^ The study was carried out according to the principles of the Declaration of Helsinki and approved by the local ethics committees. Haemodynamically stable adult patients presenting to the ED with acute chest pain/discomfort and able and willing to provide written informed consent were recruited. While enrolment was independent of renal function, patients with terminal kidney failure on chronic dialysis were excluded.

For this analysis, patients with missing data on the presence of accompanying dyspnoea, and those with an unknown diagnosis after final adjudication and at least one elevated hs-cTnT concentration possibly indicating AMI, were excluded. For the secondary analysis including the diagnostic performance of high-sensitivity cardiac troponin (hs-cTn) and the European Society of Cardiology (ESC) 0/1h-algorithms, patients with ST-segment elevation myocardial infarction (STEMI) and those with missing blood samples or hs-cTnT and hs-cTnI measurements at 1 h were excluded. The most common reasons for missing samples after 1 h were early transfer to the catheter laboratory or coronary care unit and diagnostic procedures that precluded blood draws around the 1 h window. The authors designed the study, gathered, and analysed the data according to the STrengthening the Reporting of OBservational studies in Epidemiology guidelines^24^ for observational studies in epidemiology (see Supplementary material online, *Table S1*), vouched for the data and analysis, wrote the paper, and decided to submit it for publication.

### Assessment of accompanying dyspnoea

Study staff, mostly physicians, prospectively and systematically evaluated the presence or absence of dyspnoea as an additional symptom accompanying acute chest pain/discomfort as a binary variable (yes/no) and documented on a standardized study-specific case report form while assessing the patient in the ED.^21,22,25–28^

### Centrally adjudicated final diagnosis

Two independent cardiologists performed the central adjudication of the final diagnosis applying current guidelines^3^ and the universal definition of myocardial infarction (MI)^29^ using two sets of data: first, all available medical records obtained during clinical care including history, physical examination, and results of laboratory testing including serial clinical (hs)-cTn levels, radiologic testing, ECG, echocardiography, cardiac exercise test, lesion severity and morphology in coronary angiography, and cardiac magnetic resonance imaging—pertaining to the patient from the time of ED presentation to 90-day follow-up; and second, study-specific assessments including detailed chest pain characteristics using 34 predefined criteria, serial hs-cTnT blood concentrations obtained from study samples, and clinical follow-up by telephone and/or mail. In situations of disagreement about the diagnosis, cases were reviewed and adjudicated in conjunction with a third cardiologist.

AMI was defined and (hs)-cTn was interpreted as recommended in the current guidelines, and as described in detail elsewhere.^3,29^ Similarly, details of blood sampling, biobanking, and measurements of hs-cTnT (Elecsys) and hs-cTnI (Architect) have been reported previously.^19–23^ The details of the ESC hs-cTnT/I-0/1h-algorithms are described in the online supplemental.

### Follow-up and clinical endpoints

Patients were contacted at 3, 12, and 24 months after discharge by telephone calls or in written form. We obtained information regarding death during follow-up from the patient’s hospital records, the family physician’s records and the national death registry. The primary diagnostic endpoint was the association of accompanying dyspnoea with final adjudicated diagnoses of patients. The second diagnostic endpoint was the performance of hs-cTn and the ESC 0/1h-algorithms in patients with vs. without dyspnoea quantified by the safety for rule-out, accuracy for rule-in, and overall efficacy. The primary prognostic endpoint was two-year all-cause mortality.

### Statistical analysis

Final differential diagnoses of patients with vs. without accompanying dyspnoea are depicted in bar charts. We constructed boxplots to assess and visualize differences in hs-cTnT and hs-cTnI concentrations at presentation between patients with vs. without accompanying dyspnoea in patients with non-ST-segment elevation myocardial infarction (NSTEMI) compared to patients with other final diagnoses. We further constructed receiver-operating-characteristics (ROC) curves and calculated corresponding areas under the curve (AUC) to assess the discriminative performance of hs-cTnT and hs-cTnI concentrations at presentation to diagnose NSTEMI in patients with vs. without accompanying dyspnoea. We also assessed the AUC for the clinical judgment of the likelihood for an acute coronary syndrome (ACS) as assessed by the treating physician in the ED based on the clinical examination, the ECG, and the first clinical (hs)-cTn measurement. AUCs of independent ROC curves were compared as recommended by Hanley *et al*.^30^ The diagnostic performance of the ESC 0/1h-algorithms was assessed by safety for rule-out [quantified by the resulting sensitivity and negative predictive value (NPV)], accuracy for rule-in [quantified by the resulting specificity and positive predictive value (PPV)], and overall efficacy (quantified by the percentage of patients triaged either towards rule-out or rule-in). We used cross tables derived by the application of the recommended assay-specific cut-off criteria for rule-out or rule-in of MI to calculate diagnostic performance parameters and 95% confidence intervals (95%CI) using the Wilson score method without continuity correction. Specificity, PPV, sensitivity, and NPV between independent groups were compared using Fisher’s exact test. We performed a univariable and multivariable cox proportional hazard model including common confounders which are available at presentation of patients to the ED. We calculated crude as well as adjusted hazard ratios (HRs) and created a forest plot to assess the prediction of accompanying dyspnoea of two-year cardiovascular and all-cause mortality. Cumulative all-cause mortality during two years of follow-up according to presence or absence of accompanying dyspnoea was plotted in Kaplan–Meier curves, and the log-rank test was used to assess differences in survival between groups. Continuous variables are described as median with interquartile range (IQR) and categorical variables by numbers and percentages. Continuous variables were compared with the Mann–Whitney *U* test, and categorical variables using the Pearson *X^2^* test or Fisher’s exact test, as appropriate.

All hypothesis testing was two-tailed, and *P* values of less than 0.05 were considered to indicate statistical significance without adjustments for multiple testing. Statistical analyses were performed using SPSS for Mac, version 28.0 (SPSS Inc., Chicago, IL, USA) and R version 4.1.2 (Vienna, Austria).

## Results

### Study cohort and characteristics of patients

From April 2006 to April 2018, 6684 patients were prospectively enrolled. Overall, 100 patients had missing information on accompanying dyspnoea. 6045 patients were eligible for the primary analysis, 4734 patients for the secondary analysis using the ESC hs-cTnT 0/1h-algorithm, and 4586 patients for the secondary analysis using the ESC hs-cTnI 0/1h-algorithm (see Supplementary material online, *Figure S1).* Patients with accompanying dyspnoea were older, more often female, and more often had known cardiovascular risk factors and known coronary artery disease (*Table 1*). Baseline characteristics of patients that were excluded due to missing information on dyspnoea overall were comparable to those patients in the final analysis (see Supplementary material online, *Table S2*).

**Table 1:** Baseline characteristics of the patients

	All patients (*n* = 6045)	Dyspnoea (*n* = 2892)	No dyspnoea (*n* = 3153)	*P* value
Age—years	61 (49–74)	62 (49–75)	60 (49–72)	<0.001
Female gender—no. (%)	2026 (34)	1109 (38)	917 (29)	<0.001
Risk factors—no. (%)				
ȃHypertension	3606 (60)	1822 (63)	1784 (57)	<0.001
ȃHypercholesterolaemia	2859 (47)	1405 (49)	1454 (46)	0.055
ȃDiabetes	1061 (18)	558 (19)	503 (16)	0.001
ȃCurrent smoking	1527 (25)	743 (26)	784 (25)	0.460
ȃHistory of smoking	2216 (37)	1067 (37)	1149 (36)	0.715
History—no. (%)				
ȃCoronary artery disease	1918 (32)	977 (34)	941 (30)	0.001
ȃPrevious MI	1371 (23)	690 (24)	681 (22)	0.036
ȃPrevious revascularization	1615 (27)	800 (28)	815 (26)	0.111
ȃPeripheral artery disease	319 (5)	166 (6)	153 (5)	0.123
ȃPrevious stroke	317 (5)	181 (6)	136 (4)	0.001
ȃPositive family history for CAD	1682 (31)	828 (32)	854 (30)	0.166
ECG findings—no. (%)				
ȃLeft bundle branch block	222 (4)	127 (4)	95 (3)	0.004
ȃST-segment elevation	274 (5)	125 (4)	149 (5)	0.451
ȃST-segment depression	715 (12)	376 (13)	339 (11)	0.007
ȃT-wave inversion	761 (13)	393 (14)	368 (12)	0.025
ȃNo significant ECG abnormalities	4353 (72)	2051 (71)	2302 (73)	0.071
ȃAtrial fibrillation/flutter at presentation	412 (7)	253 (9)	159 (5)	<0.001
Body mass index (kg/m^2^)	27 (24–30)	27 (24–30)	26 (24–29)	0.010
Laboratory findings				
ȃCreatinine clearance, mL/min/m^2^	87 (70–101)	86 (67–101)	88 (72–101)	<0.001
ȃHs-cTnT, ng/L	8.1 (4.0–21)	9.0 (4.0–26)	8.0 (4.0–19)	<0.001
ȃHs-cTnI, ng/L	4.5 (2.0–19)	5.0 (2.0–22)	4.1 (2.0–15)	0.001
ȃNT-proBNP^a^, ng/L	135 (43–485)	168 (50–720)	120 (34–366)	<0.001
ȃBNP^b^, ng/L	82 (29–217)	101 (31–305)	71 (26–165)	<0.001
Vital signs				
ȃBlood pressure systolic, mmHg	140 (125–156)	138 (124–155)	140 (127–157)	<0.001
ȃBlood pressure diastolic, mmHg	80 (71–90)	80 (70–90)	81 (72–91)	<0.001
ȃHeart rate, beats per minute	76 (66–89)	77 (67–90)	75 (66–88)	<0.001
ȃRespiratory rate, per minute	16 (14–20)	17 (14–20)	16 (14–19)	<0.001
ȃOxygen saturation, %	98 (97–99)	98 (96–100)	98 (97–99)	0.028
Procedures performed—no. (%)				
ȃCoronary angiography	1642 (27)	806 (28)	836 (27)	0.237
ȃPCI	1002 (17)	464 (16)	538 (17)	0.287
ȃCABG	129 (2)	65 (2)	64 (2)	0.558
Medication at presentation—no. (%)				
ȃAspirin/thienopyridine	2270 (38)	1150 (40)	1120 (36)	<0.001
ȃB-blockers	2001 (33)	1014 (35)	987 (31)	0.002
ȃStatins	2100 (35)	1037 (36)	1063 (34)	0.080
ȃACEIs/ARBs	2365 (39)	1178 (41)	1187 (38)	0.014
ȃCalcium antagonists	902 (15)	473 (16)	429 (14)	0.003
ȃNitrates	561 (9)	328 (11)	233 (7)	<0.001

Numbers are presented as median (IQR) or numbers (%).

CAD, coronary artery disease; ECG, electrocardiogram; hs-cTn, high-sensitivity cardiac troponin; NT-proBNP, N-terminal pro B-type natriuretic peptide; BNP, B-type natriuretic peptide; PCI, percutaneous coronary intervention; CABG, coronary artery bypass grafting; ACEIs, angiotensin-converting-enzyme inhibitors; ARBs, angiotensin receptor blockers.

Available in 1095 patients.

Available in 1434 patients.

### Adjudicated final diagnosis

The adjudicated final diagnosis was NSTEMI in 1339/6045 patients (22%), unstable angina (UA) in 501/6045 (8%), cardiac symptoms of origin other than coronary artery disease such as tachyarrhythmia, Takotsubo syndrome, heart failure, or myocarditis in 763/6045 (13%), non-cardiac symptoms in 3263/6045 (54%), and unknown in 179/6045 patients (3%).

### Association of accompanying dyspnoea with differential diagnoses

**Figure 1:**
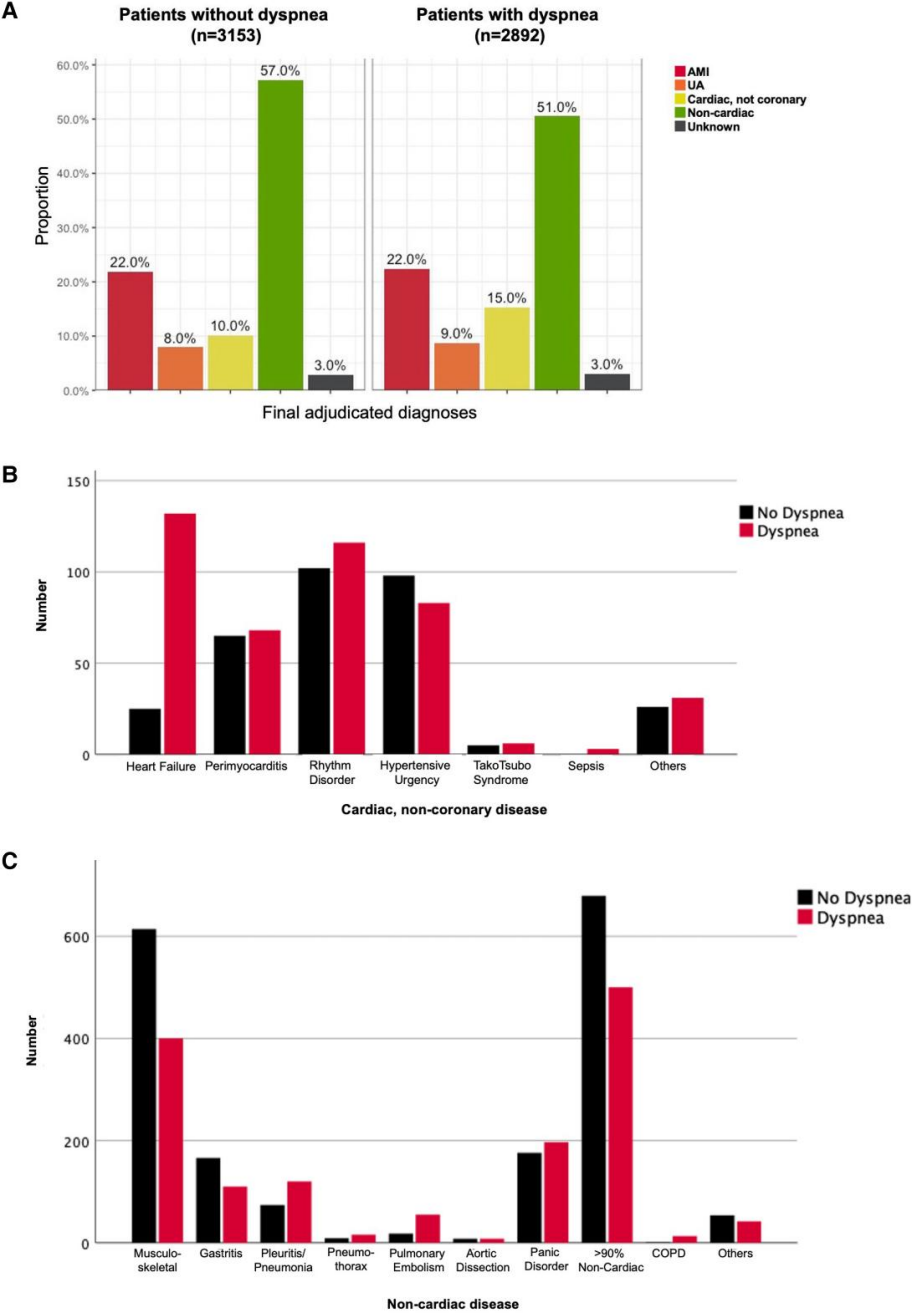
(*A*) Differential diagnoses according to the presence or absence of accompanying dyspnoea. AMI, acute myocardial infarction; UA, unstable angina. (*B*) Differential diagnoses of patients with cardiac non-coronary disease according to the presence or absence of accompanying dyspnoea. (*C*) Differential diagnoses of patients with non-cardiac disease according to the presence or absence of accompanying dyspnoea. COPD, chronic obstructive pulmonary disease.

Overall, differential diagnoses among patients with vs. without accompanying dyspnoea differed (*P* < 0.001). While the prevalence of NSTEMI including type 1 MI and type 2 MI subtypes as well as UA were comparable, cardiac, non-coronary disease was more common (15.3% vs. 10.2%), and non-cardiac disease (50.5% vs. 57.2%) less common in patients with accompanying dyspnoea vs. those without (*Figure 1A*). Among those with cardiac non-coronary disease, patients with accompanying dyspnoea had a much higher prevalence of adjudicated heart failure (30% vs. 7.8%, *P* < 0.001, *Figure 1B*). Patients with non-cardiac conditions and accompanying dyspnoea more often had pulmonary disease such as pneumonia and chronic obstructive pulmonary disease and less often musculoskeletal chest pain (*Figure 1C*).

### Hs-cTnT and hs-cTnI concentrations at presentation according to final diagnoses

**Figure 2:**
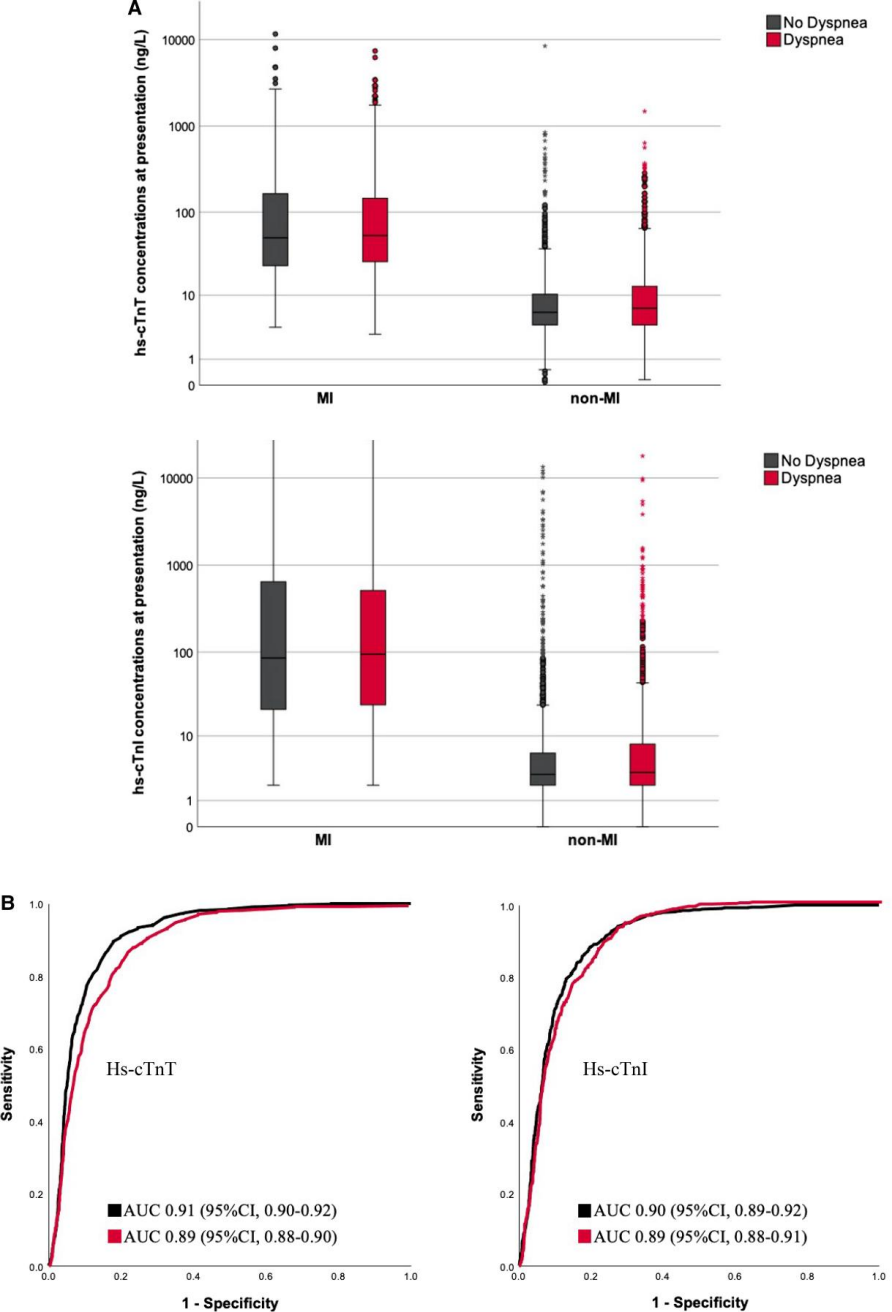
(*A*) Boxplots showing hs-cTnT and hs-cTnI concentrations according to the presence of accompanying dyspnoea in patients with MI vs. those with other adjudicated diagnoses. Boxplots showing hs-cTnT concentrations (upper boxplots) and hs-cTnI concentrations (lower boxplots) at presentation according to final diagnoses (MI vs. non-MI) in patients with vs. without accompanying dyspnoea. MI, myocardial infarction. (*B*) Diagnostic accuracy of hs-cTnT and hs-cTnI concentrations at presentation for the diagnosis of NSTEMI according to the presence or absence of accompanying dyspnoea. Receiver-operating-characteristics curves indicating the diagnostic accuracy of hs-cTnT (left) and hs-cTnI (right) concentrations at presentation for the diagnosis of NSTEMI in patients with (red line) vs. without (black line) accompanying dyspnoea. Hs-cTn, high-sensitivity cardiac troponin; AUC, area under the curve; CI, confidence interval.

In patients with NSTEMI, hs-cTnT concentrations at presentation were comparable in patients with vs. without dyspnoea [53 ng/L (IQR 26–145) vs. 50 ng/L (IQR 23–164), *P* = 0.35]. Similarly, hs-cTnI concentrations did not differ [95 ng/L (IQR 24–515) vs. 86 ng/L (IQR 21–644), *P* = 0.67]. In contrast, in patients with final diagnoses other than MI, hs-cTnT and hs-cTnI concentrations were higher in those with vs. without accompanying dyspnoea [7 ng/L (IQR 4–13) vs. 6 ng/L (IQR 4–10) for hs-cTnT and 3.2 ng/L (IQR 2–8) vs. 3 ng/L (IQR 2–6) for hs-cTnI, both *P* < 0.001, *Figure 2A*].

### B-type natriuretic peptides at presentation

B-type natriuretic peptide (BNP) as well as N-terminal (NT)-pro B-type natriuretic peptide (NT-proBNP) concentrations at presentation were higher in patients with vs. without accompanying dyspnoea [BNP 101 ng/L (IQR 31–305) vs. 71 ng/L (IQR 26–165) and NT-proBNP 168 ng/L (IQR 50–720) vs. 120 ng/L (IQR 34–366), respectively, both *P* < 0.001].

### Diagnostic accuracy of hs-cTnT and hs-cTnI at presentation

Diagnostic accuracy of hs-cTnT for NSTEMI was similar in patients with vs. without accompanying dyspnoea with AUCs of 0.89 (95%CI, 0.88–0.90) and 0.91 (95%CI, 0.90–0.92, *P* = 0.07), respectively. Findings were similar for hs-cTnI with AUCs of 0.89 (95%CI, 0.88–0.91) and 0.90 (95%CI, 0.89–0.92, *P* = 0.44), respectively (*Figure 2B*).

### Association of dyspnoea with clinical judgment, time in the emergency department, and rate of hospitalization

The clinical judgment for the presence of an ACS, as assessed by the treating physician in the ED, was similar in patients with vs. without accompanying dyspnoea [30% (95%CI, 10–70) vs. 30% (10–60)]. The AUC of the clinical judgment for an ACS was 0.85 (95%CI, 0.84–0.87) in patients with vs. 0.87 (95%CI, 0.86–0.89) in patents without dyspnoea (*P* = 0.07). Patients with accompanying dyspnoea spent more time in the ED than patients without [280 min (IQR 177–415) vs. 268 min (IQR 165–401); *P* = 0.004; Supplementary material online, *Figure S2*] and were more likely to be hospitalized (41% vs. 32%; *P* < 0.001).

### Diagnostic performance of the European Society of Cardiology hs-cTn 0/1h-algorithms

**Figure 3:**
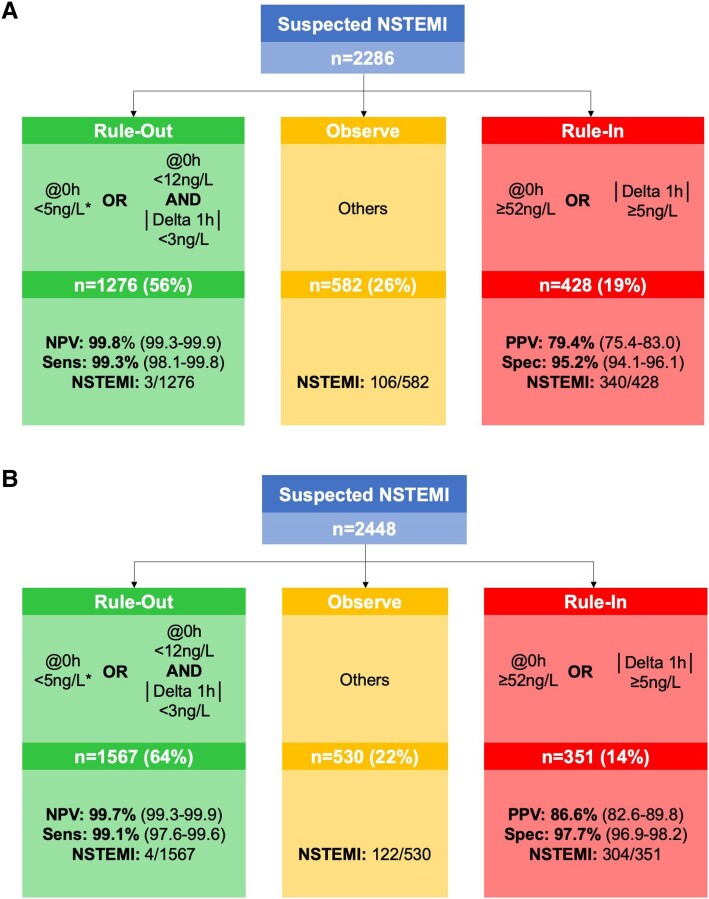

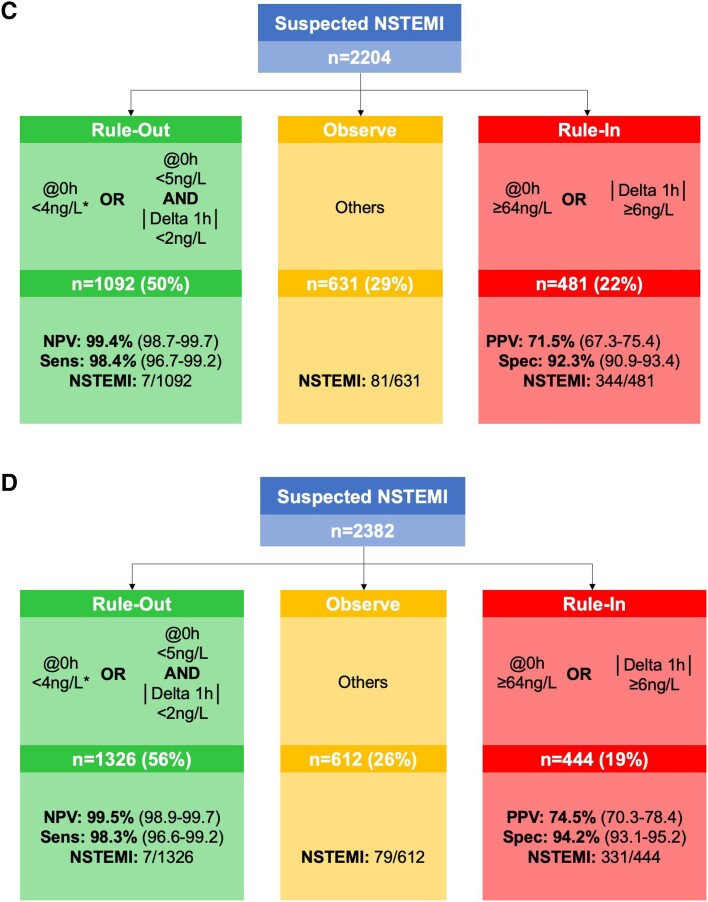
(*A+B*) Diagnostic performance of the ESC hs-cTnT 0/1h-algorithm. Diagnostic performance of the ESC hs-cTnT 0/1h-algorithm in (*A*) patients with and (*B*) patients without accompanying dyspnoea. *, if chest pain onset >3 h; Delta, unsigned change within the first hour; NSTEMI, non-ST-segment elevation myocardial infarction; Sens., sensitivity; NPV, negative predictive value; Prev., prevalence; Spec., specificity; PPV, positive predictive value; hs-cTnT, high-sensitivity cardiac troponin T. (*C+D*) Diagnostic performance of the ESC hs-cTnI 0/1h-algorithm. Diagnostic performance of the ESC hs-cTnI 0/1h-algorithm in (*C*) patients with and (*D*) patients without accompanying dyspnoea. *, if chest pain onset >3 h; Delta, unsigned change within the first hour; NSTEMI, non-ST-segment elevation myocardial infarction; Sens., sensitivity; NPV, negative predictive value; Prev., prevalence; Spec., specificity; PPV, positive predictive value; hs-cTnI, high-sensitivity cardiac troponin I.

Among 2286 patients with dyspnoea, the ESC hs-cTnT 0/1h-algorithm ruled-out 1276 patients (56%) with a sensitivity of 99.3% (95%CI, 98.1–99.8) and a NPV of 99.8% (95%CI, 99.3–99.9). On the other hand, 428 patients (19%) were ruled-in with a specificity of 95.2% (95%CI, 94.1–96.1) and a PPV of 79.4% (95%CI, 75.4–83.0). The remaining 582 patients (26%) were triaged towards observe with a NSTEMI prevalence of 18% (*Figure 3A*).

Among 2448 patients without dyspnoea, the ESC hs-cTnT 0/1h-algorithm ruled-out 1567 patients (64%) with a sensitivity of 99.1% (95%CI, 97.6–99.6) and a NPV of 99.7% (95%CI, 99.3–99.9). On the other hand, 351 patients (14%) were ruled-in with a specificity of 97.7% (95%CI, 96.9–98.2) and a PPV of 86.6% (95%CI, 82.6–89.8). The remaining 530 patients (22%) were triaged towards observe with a NSTEMI prevalence of 23% (*Figure 3B*). While the safety for rule-out was similar in patients with vs. without dyspnoea (*P* = 0.72 and *P* = 1.0 for differences in sensitivity and NPV), the rule-in accuracy was lower in patients with dyspnoea (*P* < 0.001 and *P* = 0.01 for differences in specificity and PPV, respectively). Findings for the ESC hs-cTnI 0/1h-algorithm were comparable (*P* = 0.018 for difference in PPV; *Figure 3*C+D).

### Cox proportional hazards regression analysis

**Figure 4:**
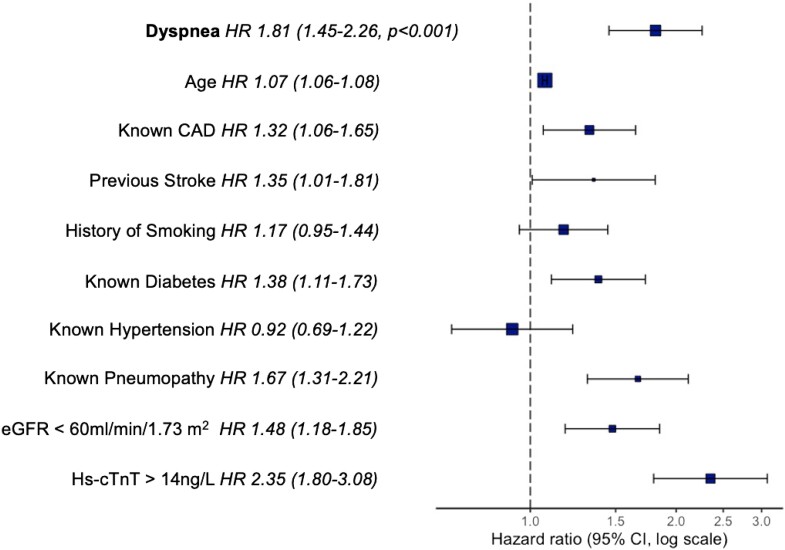
Forest plot showing adjusted hazard ratios from multivariable cox regression analysis. Hazard ratios from multivariable cox proportional hazard model for all-cause death at 2 years. HR, hazard ratio; CAD, coronary artery disease; eGFR, estimated glomerular filtration rate; hs-cTnT, high-sensitivity cardiac troponin T.

In univariable cox regression analyses, accompanying dyspnoea was associated with cardiovascular and all-cause mortality at two years [unadjusted HRs 2.243 (1.703–2.954) and 2.487 (95%CI, 2.001–3.091), respectively, both *P* < 0.001]. In multivariable cox regression models, after adjustment for other significant predictors of outcome including cardiovascular risk factors as well as confounders that are available at presentation, accompanying dyspnoea remained an independent predictor for cardiovascular and all-cause mortality at two years of follow-up [adjusted HRs 1.576 (95%CI, 1.193–2.083) and 1.813 (95%CI, 1.453–2.261), respectively, both *P* < 0.01; *Table 2*, *Figure 4*].

**Table 2:** Cox regression analysis for cardiovascular death at two years of follow-up

	Univariable unadjusted hazard ratio (95%CI) *P* value	Multivariable adjusted hazard ratio (95%CI) *P* value
Dyspnoea	2.243 (1.703–2.954)	1.576 (1.193–2.083)
	<0.001	0.001
Age	1.107 (1.093–1.121)	1.072 (1.056–1.088)
	<0.001	<0.001
Sex	1.112 (0.849–1.458)	—
	0.441	
Known CAD	3.575 (2.736–4.670)	1.520 (1.144–2.021)
	<0.001	0.004
Previous stroke	3.651 (2.567–5.191)	1.450 (1.007–2.086)
	<0.001	0.046
History of smoking	1.558 (1.201–2.021)	0.928 (0.709–1.216)
	<0.001	0.589
Known diabetes	2.424 (1.840–3.192)	1.361 (1.022–1.811)
	<0.001	0.035
Known arterial hypertension	4.071 (2.815–5.888)	0.958 (0.652–1.407)
	<0.001	0.825
Known pneumopathy	2.954 (2.181–4.001)	1.612 (1.182–2.198)
	<0.001	0.003
GFR < 60 mL/min/1.73 m^2^	6.864 (5.285–8.914)	1.670 (1.252–2.227)
	<0.001	<0.001
High-sensitivity cardiac troponin > 14 ng/L (99th centile)	10.030 (7.105–14.159)	3.069 (2.107–4.471)
	<0.001	<0.001

CAD, coronary artery disease; GFR, glomerular filtration rate; b.p.m., beats per minute.

### Cardiovascular and all-cause mortality at two years of follow-up

**Figure 5:**
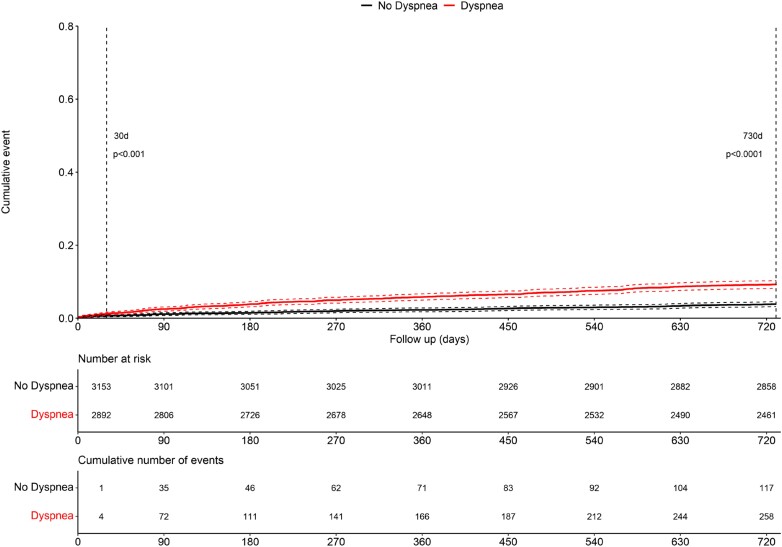
Cumulate death at 30 days and two years of follow-up in patients with vs. without accompanying dyspnoea. Kaplan–Meier curves indicating cumulative all-cause death at 30 days and two years of follow-up in patients without (black line) vs. with (red line) accompanying dyspnoea. Differences between survival rates were assessed using the log-rank test.

Patients with accompanying dyspnoea had higher cardiovascular [152/2892 (5.3%) vs. 76/3153 (2.4%)] and all-cause mortality [261/2892 (9.0%) vs. 118/3153 (3.7%)] rates at two years of follow-up than patients without dyspnoea (both *P* < 0.001; *Figure 5*).

## Discussion

We assessed the impact of accompanying dyspnoea on final diagnoses, on diagnostic performance of hs-cTn and the ESC 0/1h-algorithms, and on the outcome of patients presenting with acute chest pain/discomfort to the ED. We report six major findings: first, chest pain/discomfort patients with accompanying dyspnoea were slightly older, more often female, and more often had arterial hypertension and diabetes. Second, accompanying dyspnoea was not associated with a higher ACS prevalence. However, patients with dyspnoea more often had cardiac, non-coronary (15.3% vs. 10.1%), and less often non-cardiac disease (50.5% vs. 57.2%). Third, hs-cTnT and hs-cTnI concentrations at presentation were similar in patients with NSTEMI irrespective of the presence of accompanying dyspnoea. In contrast, patients with other final diagnoses than NSTEMI and accompanying dyspnoea had slightly (10–20%) higher hs-cTnT and hs-cTnI concentrations than those without dyspnoea. This difference may be explained by the fact that patients with accompanying dyspnoea more often had cardiac, non-coronary disease, such as heart failure, which often is associated with acute and chronic myocardial injury. Furthermore, both BNP and NT-proBNP concentrations at presentation were higher in patients with vs. without accompanying dyspnoea, reflecting underlying cardiac disease and increased intracardiac filling pressures. Fourth, while overall the diagnostic accuracy of hs-cTnT/I concentrations for NSTEMI and the early clinical judgment for the presence of an ACS were very high also in patients with accompanying dyspnoea (AUC 0.89 for hs-cTnT, 0.90 for hs-cTnI, and 0.85 for clinical judgment, respectively), these patients stayed slightly longer in the ED and were much more likely to require hospitalisation (41% vs. 32%) vs. patients without accompanying dyspnoea. Fifth, while the high safety of the ESC 0/1h-algorithms was maintained in patients with accompanying dyspnoea with very high NPV and sensitivity, the specificity and PPV for rule-in slightly decreased in patients with dyspnoea [specificity 95.2% (95%CI, 94.1–96.1) vs. 97.7% (95%CI, 96.9–98.2), *P* < 0.001; PPV 79.4% (95%CI, 75.4–83.0) vs. 86.6% (95%CI, 82.6–89.8), *P* = 0.01]. The presence of dyspnoea affected the overall performance of the ESC 0/1h-algorithms as fewer patients were triaged towards rule-out and slightly more patients towards rule-in. Consequently, more patients remained in the observe zone. As dyspnoea was associated with higher hs-cTn concentrations due to other underlying cardiac disease than MI, these findings are well in line with previous studies investigating the performance of the ESC 0/1h-algorithms in the elderly and those with renal dysfunction.^21,31,32^ Sixth, after adjustment for other significant predictors of outcome, dyspnoea remained an independent predictor for cardiovascular and all-cause mortality at two years of follow-up with HRs of 1.576 (95%CI, 1.193–2.083) and 1.813 (1.453–2.261, both *P* < 0.01), respectively. Furthermore, patients with accompanying dyspnoea were at much higher risk for cardiovascular and all-cause mortality at two years.

These findings confirm and extend prior work in which dyspnoea was assessed as an alternative symptom to chest pain/discomfort.^33,34^ Among 592 dyspnoeic patients presenting to the ED, clinical uncertainty, as assessed by the treating ED physician, was present in 185 (31%) patients, with a low diagnostic accuracy of clinical judgment (AUC 0.76, 95%CI, 0.69–0.83) for the presence of acute heart failure. Clinical uncertainty was an independent predictor of death.^34^ In a secondary analysis from the multicentre randomized controlled PROMOTION (Patient Response tO Myocardial Infarction fOllowing a Teaching Intervention Offered by Nurses) trial, differences in symptoms leading to ED presentations among 3522 patients with known CAD were investigated.^17^ At two years of follow-up, 234 patients presented with non-ACS vs. 331 patients with ACS. Dyspnoea was present in 33% vs. 25% (*P* = 0.028), respectively, and therefore more prevalent in patients with non-ACS. Also in the prehospital setting, dyspnoea was found to be predominantly present in elderly female patients and associated with significantly increased mortality rates compared to patients presenting with chest pain [13% (IQR 12–15) vs. 2.9% (2.6–3.2) at 30 days and 50% (IQR 47–54) vs. 20% (IQR 19–21) at 4 years].^35^ In a large series of patients referred for myocardial-perfusion single-photon-emission computed tomography, self-reported dyspnoea identified a subgroup of otherwise asymptomatic patients at increased risk for death from cardiac or any cause irrespective of the presence of known CAD. Since the authors only coded dyspnoea among patients without chest pain, they could not evaluate the potential interaction between dyspnoea and symptoms of chest pain. Although we have investigated the impact of dyspnoea in a different clinical setting, most of our findings are well in line with those reported with a nearly identical HR for all-cause death at 2 years.^33^

Our results indicate that accompanying dyspnoea in chest pain/discomfort patients is an important symptom and imply that when dyspnoea is present, the risk of death from any cause is increased. Accordingly, it seems appropriate to include an evaluation of dyspnoea in the clinical assessment of patients presenting with chest pain/discomfort to the ED and to evaluate whether a further diagnostic work-up is indicated to identify and potentially treat the underlying cause, such as heart failure, pulmonary embolism, or COPD.

Our findings do also extend data previously obtained for the diagnostic performance of the ESC 0/1h-algorithm assessed in all-comers with acute chest discomfort.^7,8,10,36^ The safety of the ESC 0/1h-algorithms remained very high in patients with accompanying dyspnoea. However, one has to consider that the overall performance was slightly impaired in patients with dyspnoea due to the higher prevalence of chronic and acute myocardial injury, similarly to patients with kidney failure or the elderly, resulting in lower PPV for rule-in and a higher number of patients remaining in the observe zone.^21,31,32^

Some limitations merit consideration when interpreting these findings. First, our study was conducted in ED patients with symptoms suggestive of MI. Further studies are required to quantify the impact of accompanying dyspnoea in chest pain patients with a higher pre-test probability (e.g. in a coronary care unit setting) or in patients with a lower pre-test probability (e.g. in a general practitioner setting) for MI. Second, no specific sample size calculation was performed. Although this secondary analysis from an ongoing multicentre study is one of the largest ever performed, it still may have been underpowered for some comparisons. Third, not all patients with acute chest pain had a second set of laboratory measurements at 1 h. The most common reasons for missing blood samples were logistic issues in the ED that precluded blood draw around the 1 h window. However, it is unlikely that the absence of these patients significantly influenced our results. Fourth, although we used the most stringent methodology to adjudicate the presence or absence of MI including central adjudication by experienced cardiologists and serial measurements of hs-cTn, we still may have misclassified a small number of patients. Fifth, as the presence of dyspnoea was self-reported, we cannot exclude that the prevalence of underlying conditions might be slightly under- or overestimated. Sixth, BNP and NT-proBNP concentrations at presentation were only available in a subset of patients. Furthermore, we did not systematically assess left and right ventricular function and intracardiac pressures to answer the question whether heart failure might have been the major driver of higher mortality rates in patients with dyspnoea. Finally, we cannot generalize our findings to patients with terminal kidney failure requiring dialysis, since they were excluded from this study.

## Conclusion

Accompanying dyspnoea was not associated with a higher prevalence of ACS but with cardiac, non-coronary disease. While the safety of the diagnostic work-up was not affected, accompanying dyspnoea was an independent predictor for cardiovascular and all-cause death.

## Supplementary material

Supplementary material is available at *European Heart Journal: Acute Cardiovascular Care* online.

## Supplementary Material

zuad026_Supplementary_Data

## Data Availability

The data of this study is not publicly available.
