# Supplementary material for: Association of accompanying dyspnoea with diagnosis and outcome of patients presenting with acute chest discomfort
Source: Eur Heart J Acute Cardiovasc Care. 2023 Mar 14;12(5):283–95. doi: 10.1093/ehjacc/zuad026 (PMC10243981; doi:10.1093/ehjacc/zuad026)
Supplement: zuad026_Supplementary_Data [file zuad026_supplementary_data.docx]

# Association of accompanying dyspnea with diagnosis and outcome of patients presenting with acute chest discomfort

Online Supplemental

**Supplemental Methods**

1. Adjudication of the final diagnosis
2. Clinical Care: The (hs)-cTn assays and cut-off levels used for local clinical care
3. Central adjudication: Definition of rise and/or fall in high-sensitivity cardiac troponin T (hs-cTnT)
4. The ESC hs-cTn 0/1h-algorithm

**Supplemental Tables**

1. Supplemental Table 1 – STROBE checklist
2. Supplemental Table 2 – Baseline characteristics of patients with missing information on dyspnea

**Supplemental Figures**

1. Supplemental Figure 1 – Patient flow diagram
2. Supplemental Figure 2 – Cumulative density plot for time spent in Emergency Department

**Supplemental Methods**

**Adjudication of the final diagnosis**

AMI was defined and cTn levels interpreted as recommended in current guidelines.^1,2^ In brief, AMI was diagnosed when there was evidence of myocardial necrosis with a significant rise and/or fall in a clinical setting consistent with myocardial ischemia. Patients with AMI were further subdivided into type 1 AMI (primary coronary events) and type 2 AMI (ischemia due to increased demand or decreased supply, for example tachyarrhythmia or hypertensive crisis).^2,3^

The adjudication of final diagnoses was performed centrally in the core lab (University Hospital Basel) for all patients incorporating levels of hs-cTnT (see test characteristics in the main body of the manuscript). More specifically, two independent cardiologists not directly involved in patient care reviewed all available medical records (including patient history, physical examination, results of laboratory testing including hs-cTnT levels, radiologic testing, ECG, echocardiography, cardiac exercise test, lesion severity and morphology in coronary angiography, discharge summary) pertaining to the patient from the time of ED presentation to 90-day follow-up. Late samples were available for adjudication of final diagnosis in all patients. In general, serial sampling was performed until at least 6h after presentation to the ED or onset of chest pain. In situations of diagnostic disagreement, cases were reviewed and adjudicated in conjunction with a third cardiologist.

The 99^th^ percentile (14ng/L) was used as cut-off for myocardial necrosis. Absolute cTn changes were used to determine significant changes based on the diagnostic superiority of absolute over relative changes.^4–9^ Based on studies of the biological variation of cTn^10,11^ as well as on data from previous chest pain cohort studies,^4,12^ a significant absolute change was defined as a rise or fall of at least 10ng/L within six hours, or, in an assumption of linearity, as an absolute change of 6ng/L within three hours. Predefined alternative diagnoses included “unstable angina” (UA), “Cardiac symptoms of origin other than coronary artery disease” and “non-cardiac chest pain”.

**Clinical Care: The (hs)-cTn assays and cut-off levels used for local clinical care**

Routine clinical care comprised five different cTn assays at the different hospitals and at the different recruitment periods. The cTn assays used clinically in most of the participating institutions changed during the study from a conventional cTn assay to the hs-cTnT assay. In order to take advantage of the higher sensitivity and higher overall diagnostic accuracy offered by the hs-cTnT assay, patients were adjudicated using the hs-cTnT values in all patients. In patients in whom clinically a conventional cTn assay was used, the conventional cTn values and the hs-cTnT values were available for the adjudication. In patients in whom clinically the hs-cTnT assay was used, only the hs-cTnT values were available for the adjudication.

The following conventional cTn assays were used: For the Roche cTnT 4^th^ generation assay, the 10% CV level is 0.035µg/l. The laboratories of the participating sites reported only two decimals; therefore 0.04µg/l was used as a cut-off for myocardial necrosis. In order to fulfil the criteria of a significant change (30% of 99^th^ percentile or 10% CV level), a patient would e.g. need to have a level of <0.01µg/l at presentation and 0.04µg/l at 6h. A patient would also qualify if the first level is 0.02µg/l and the second 0.04µg/l. A patient would not fulfil the criteria if the first level is 0.03µg/l and the second is 0.04µg/l. If the first level is 0.04µg/l, the second level needs to be at least 0.06µg/l.

For the Abbott Axsym cTnI ADV, the 10% CV level is 0.16µg/l. A patient having 0.16µg/l at presentation would meet the criteria for significant change if the second was ≥0.21µg/l. A patient having <0.12ug/l at presentation (limit of detection) would qualify if the second is >0.16µg/l.

For the Beckmann Coulter Accu cTnI, the 10% CV level is 0.06µg/l. A patient having 0.06µg/l at presentation would qualify if the second is ≥0.08µg/l. A patient having 0.05µg/l at presentation would qualify if the second is 0.07µg/l, but not 0.06µg/l. A patient having undetectable cTnI (cTnI <0.01µg/l) at presentation would qualify if the second is ≥0.06µg/l.

For the Siemens Dimension Vista s-cTnI, the 10% CV level is 40ng/L. The limit of detection is 15ng/L and the 99^th^ percentile is 45ng/L. An absolute change of 20ng/L or more within 3-6h was considered significant.

For Elecsys hs-cTnT measured clinically, the same change criteria were applied as for hs-cTnT measured from the study blood samples.

**Central adjudication: Definition of rise and/or fall in high-sensitivity cardiac troponin T (hs-cTnT)**

Absolute changes in hs-cTnT were used to determine significant changes based on the diagnostic superiority of absolute over relative changes.^4–9^ Based on studies of the biological variation of cTn^10,11^ as well as on data from previous chest pain cohort studies,^4,12^ a significant absolute change was defined as a rise or fall of at least 10ng/L within 6 hours or an absolute change of 6ng/L within 3 hours. If later clinical samples (e.g., at 24, 48, or 72 hours) revealed a lower hs-cTnT level than that measured during the period of sampling in the ED, the later level was considered the true baseline level for the calculation of the change criteria.

**The ESC hs-cTn 0/1h-algorithm**

When using hs-cTnT, patients are triaged towards rule-out, if hs-cTnT concentrations at presentation are <5ng/L and if the time since chest pain onset is >3h (direct rule-out). Alternatively, patients are ruled-out, if hs-cTnT concentrations at presentation are <12ng/L *and* if the absolute change within 1h is <3ng/L. Patients are triaged towards ruled-in for AMI, if hs-cTnT concentrations at presentation are ≥52ng/L (direct rule-in) *or* if the absolute change within 1h is ≥5ng/L. Patients fulfilling neither of the above criteria are triaged to the observe zone. When using hs-cTnI (Architect), patients are triaged towards rule-out, if hs-cTnI concentrations at presentation are <4ng/L and if the time since chest pain onset is >3h (direct rule-out). Alternatively, patients are triaged towards ruled-out, if hs-cTnI concentrations at presentation are <5ng/L *and* if the absolute change within 1h is <2ng/L. Patients are triaged towards ruled-in for AMI, if hs-cTnI concentrations at presentation are ≥64ng/L (direct rule-in) *or* if the absolute change within 1h is ≥6ng/L. Patients fulfilling neither of the above criteria are triaged to the observe zone.

**Online Tables and Figures**

|  | Item No | Recommendation | Page No |
| --- | --- | --- | --- |
| **Title and abstract** | 1 | (*a*) Indicate the study’s design with a commonly used term in the title or the abstract |  |
|  |  | (*b*) Provide in the abstract an informative and balanced summary of what was done and what was found | 1, 2 |
| Introduction | | | |
| Background/rationale | 2 | Explain the scientific background and rationale for the investigation being reported | 4 |
| Objectives | 3 | State specific objectives, including any prespecified hypotheses | 5, 6 |
| Methods | | | |
| Study design | 4 | Present key elements of study design early in the paper | 5 |
| Setting | 5 | Describe the setting, locations, and relevant dates, including periods of recruitment, exposure, follow-up, and data collection | 5-8 |
| Participants | 6 | (*a*) Give the eligibility criteria, and the sources and methods of selection of participants. Describe methods of follow-up |  |
|  |  | (*b*) For matched studies, give matching criteria and number of exposed and unexposed | 5 |
| Variables | 7 | Clearly define all outcomes, exposures, predictors, potential confounders, and effect modifiers. Give diagnostic criteria, if applicable | 6-8 |
| Data sources/ measurement | 8* | For each variable of interest, give sources of data and details of methods of assessment (measurement). Describe comparability of assessment methods if there is more than one group | 8-10 |
| Bias | 9 | Describe any efforts to address potential sources of bias | 15-16 |
| Study size | 10 | Explain how the study size was arrived at | 15-16 |
| Quantitative variables | 11 | Explain how quantitative variables were handled in the analyses. If applicable, describe which groupings were chosen and why | 7-8 |
| Statistical methods | 12 | (*a*) Describe all statistical methods, including those used to control for confounding | 7-8 |
|  |  | (*b*) Describe any methods used to examine subgroups and interactions |  |
|  |  | (*c*) Explain how missing data were addressed |  |
|  |  | (*d*) If applicable, explain how loss to follow-up was addressed |  |
|  |  | (*e*) Describe any sensitivity analyses |  |
| Results | | |  |
| 2Participants | 13* | (a) Report numbers of individuals at each stage of study—eg numbers potentially eligible, examined for eligibility, confirmed eligible, included in the study, completing follow-up, and analysed | 9-12 |
|  |  | (b) Give reasons for non-participation at each stage |  |
|  |  | (c) Consider use of a flow diagram |  |
| Descriptive data | 14* | (a) Give characteristics of study participants (eg demographic, clinical, social) and information on exposures and potential confounders | 9-12 |
|  |  | (b) Indicate number of participants with missing data for each variable of interest |  |
|  |  | (c) Summarise follow-up time (eg, average and total amount) |  |
| Outcome data | 15* | Report numbers of outcome events or summary measures over time | 11-12 |

| Main results | 16 | (*a*) Give unadjusted estimates and, if applicable, confounder-adjusted estimates and their precision (eg, 95% confidence interval). Make clear which confounders were adjusted for and why they were included | 10-13 |
| --- | --- | --- | --- |
|  |  | (*b*) Report category boundaries when continuous variables were categorized |  |
|  |  | (*c*) If relevant, consider translating estimates of relative risk into absolute risk for a meaningful time period |  |
| Other analyses | 17 | Report other analyses done—eg analyses of subgroups and interactions, and sensitivity analyses | 10-13 |
| Discussion | | | |
| Key results | 18 | Summarise key results with reference to study objectives | 14-15 |
| Limitations | 19 | Discuss limitations of the study, taking into account sources of potential bias or imprecision. Discuss both direction and magnitude of any potential bias | 16-17 |
| Interpretation | 20 | Give a cautious overall interpretation of results considering objectives, limitations, multiplicity of analyses, results from similar studies, and other relevant evidence | 16 |
| Generalisability | 21 | Discuss the generalisability (external validity) of the study results | 15-17 |
| Other information | | | |
| Funding | 22 | Give the source of funding and the role of the funders for the present study and, if applicable, for the original study on which the present article is based | 17-18 |

*Give information separately for exposed and unexposed groups.

| **Supplemental Table 1** | STROBE Statement—Checklist of items that should be included in reports of cohort studies |
| --- | --- |

| **Supplemental Table 2** | **Baseline characteristics** | | |
| --- | --- | --- | --- |
|  | Final study cohort (n=6045) | Patients with missing dyspnea  (n=100) | p-Value |
| Age – years | 61 (49-74) | 63 (51-73) | 0.464 |
| Female gender – no. (%) | 2026 (34) | 37 (37) | 0.458 |
| Risk factors – no. (%) |  |  |  |
| Hypertension | 3606 (60) | 63 (63) | 0.498 |
| Hypercholesterolemia | 2859 (47) | 50 (50) | 0.591 |
| Diabetes | 1061 (18) | 14 (14) | 0.426 |
| Current smoking | 1527 (25) | 26 (26) | 0.908 |
| History of smoking | 2216 (37) | 38 (38) | 0.782 |
| History – no. (%) |  |  |  |
| Coronary artery disease | 1918 (32) | 34 (34) | 0.628 |
| Previous MI | 1371 (23) | 22 (22) | 0.872 |
| Previous revascularization | 1615 (27) | 21 (21) | 0.212 |
| Peripheral artery disease | 319 (5) | 7 (7) | 0.373 |
| Previous stroke  Positive family history for CAD | 317 (5)  1682 (31) | 2 (2)  22 (22) | 0.175  0.472 |
| ECG findings – no. (%) |  |  |  |
| Left bundle branch block  ST-segment elevation | 222 (4)  274 (5) | 3 (3)  9 (9) | 0.723  0.049 |
| ST-segment depression | 715 (12) | 13 (13) | 0.754 |
| T-wave inversion | 761 (13) | 7 (7) | 0.125 |
| No significant ECG abnormalities  Atrial fibrillation/flutter at presentation | 4353 (72)  412 (7) | 74 (74)  2 (2) | 0.737  0.067 |
| Body mass index (kg/m^2^)  Laboratory findings | 27 (24-30) | 27 (24-30) | 0.895 |
| Creatinine clearance, mL/min/m^2^  Hs-cTnT, ng/L  Hs-cTnI, ng/L  Vital signs  Blood pressure systolic, mmHg  Blood pressure diastolic, mmHg  Heart rate, beats per minute  Respiratory Rate, per minute  Oxygen Saturation, %  Procedures performed, no. (%)  Coronary Angiography  PCI  CABG | 87 (70-101)  8.1 (4.0-21)  4.5 (2.0-19)  140 (125-156)  80 (71-90)  76 (66-89)  16 (14-20)  98 (97-99)  1642 (27)  1002 (17)  129 (2) | 87 (72-99)  9.7 (6.0-22)  4.6 (2.7-47)  148 (128-162)  81 (74-90)  76 (64-88)  17 (14-20)  98 (97-99)  29 (29)  21 (21)  2 (2) | 0.875  0.047  0.185  0.011  0.361  0.495  0.143  0.762  0.652  0.226  1.000 |

| **Supplemental Table 2 (continued)** | **Baseline characteristics** | | |
| --- | --- | --- | --- |
|  | All patients (n=6045) | Dyspnea  (n=2892) | p-Value |
| Medication at presentation – no. (%) |  |  |  |
| Aspirin/Thienopyridine | 2270 (38) | 43 (43) | 0.265 |
| B-blockers | 2001 (33) | 35 (35) | 0.689 |
| Statins | 2100 (35) | 31 (31) | 0.436 |
| ACEIs/ARBs | 2365 (39) | 38 (38) | 0.819 |
| Calcium antagonists | 902 (15) | 11 (11) | 0.322 |
| Nitrates | 561 (9) | 5 (5) | 0.164 |
|  |  |  |  |

Numbers are presented as median (IQR) or numbers (%). CAD = Coronary artery disease; ECG = Electrocardiogram; Hs-cTn = High-sensitivity cardiac troponin; PCI = Percutaneous coronary intervention; CABG = Coronary artery bypass grafting; ACEIs = Angiotensin-converting-enzyme inhibitors; ARBs = Angiotensin receptor blockers.


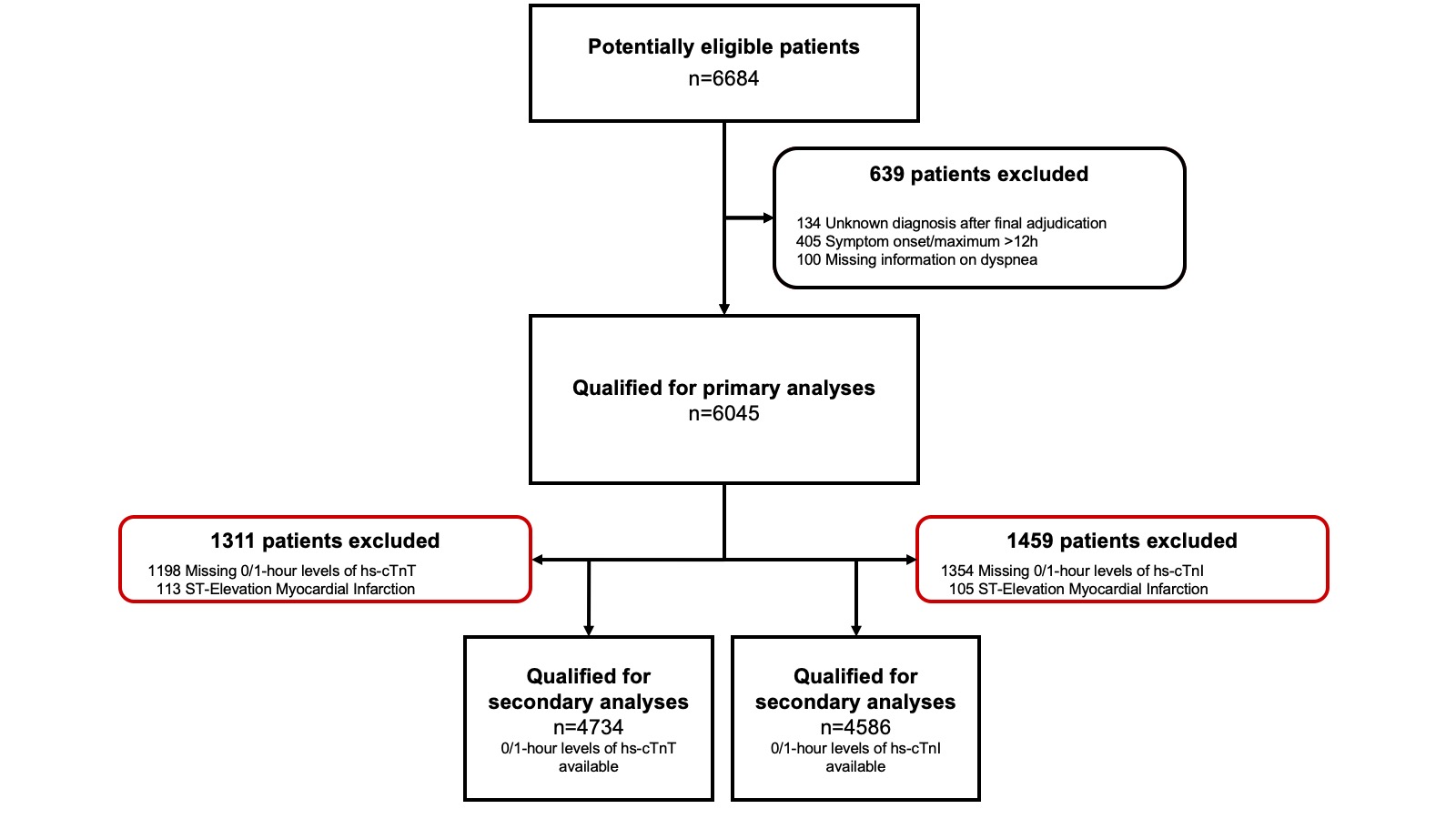


| **Supplemental**  **Figure 1** | **Patient Flow** |
| --- | --- |


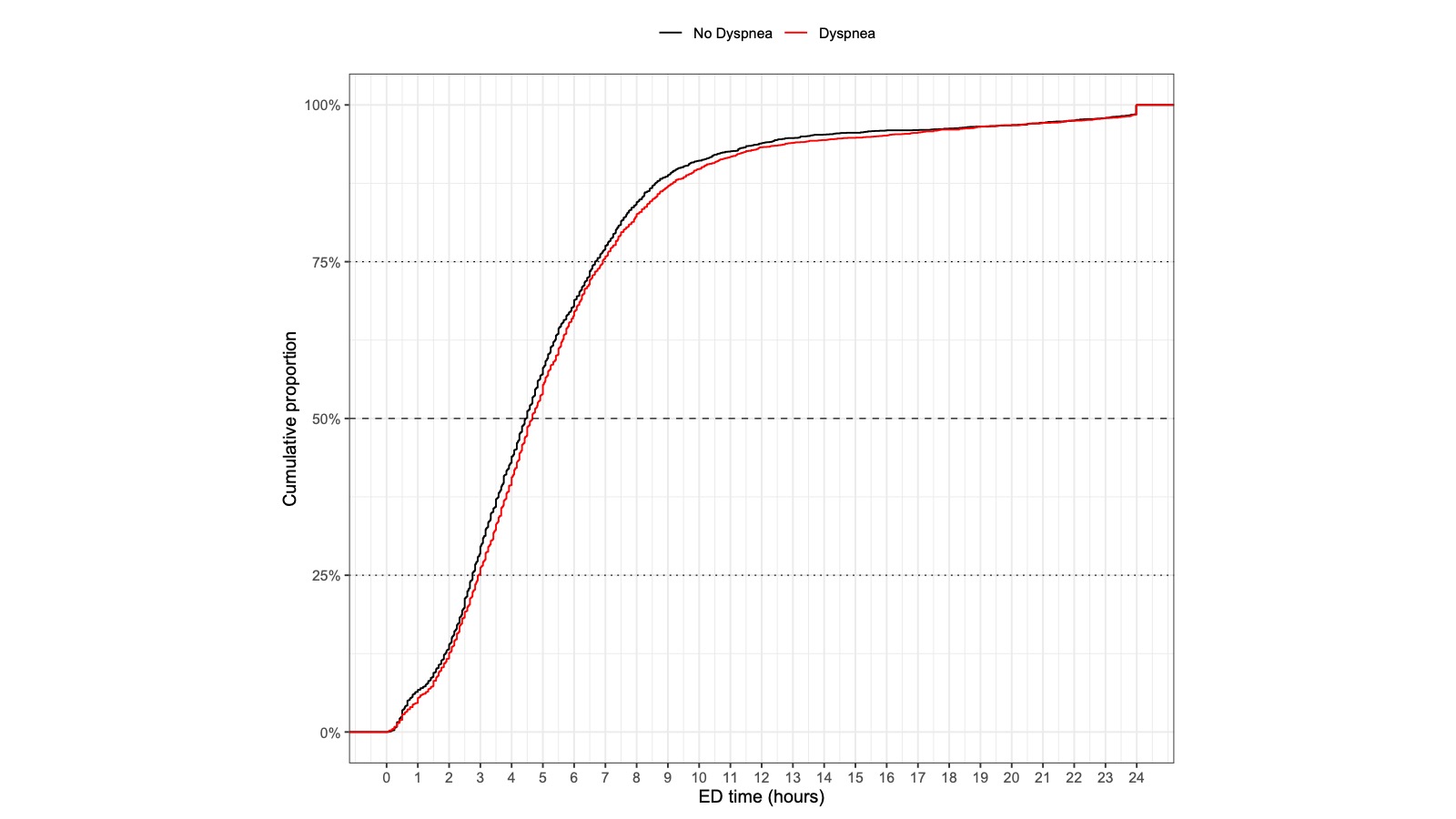


| **Supplemental**  **Figure 2** | **Cumulative density plot for time spent in Emergency Department** |
| --- | --- |

**References**

1. Apple FS, Jesse RL, Newby LK, Wu AHB, Christenson RH. National Academy of Clinical Biochemistry and IFCC Committee for Standardization of Markers of Cardiac Damage Laboratory Medicine Practice Guidelines: Analytical issues for biochemical markers of acute coronary syndromes. *Circulation* 2007;**115**:e352-5.

2. Thygesen K, Alpert JS, Jaffe AS, Chaitman BR, Bax JJ, Morrow DA, White HD. Fourth universal definition of myocardial infarction (2018). Russ. J. Cardiol. 2019. p. 107–138.

3. Collet JP, Thiele H, Barbato E, Bauersachs J, Dendale P, Edvardsen T, Gale CP, Jobs A, Lambrinou E, Mehilli J, Merkely B, Roffi M, Sibbing D, Kastrati A, Mamas MA, Aboyans V, Angiolillo DJ, Bueno H, Bugiardini R, Byrne RA, Castelletti S, Chieffo A, Cornelissen V, Crea F, Delgado V, Drexel H, Gierlotka M, Halvorsen S, Haugaa KH, Jankowska EA, et al. 2020 ESC Guidelines for the management of acute coronary syndromes in patients presenting without persistent ST-segment elevation. *Eur Heart J* 2021;**42**:1289–1367.

4. Reichlin T, Irfan A, Twerenbold R, Reiter M, Hochholzer W, Burkhalter H, Bassetti S, Steuer S, Winkler K, Peter F, Meissner J, Haaf P, Potocki M, Drexler B, Osswald S, Mueller C. Utility of absolute and relative changes in cardiac troponin concentrations in the early diagnosis of acute myocardial infarction. *Circulation* 2011;**124**:136–145.

5. Wildi K, Reichlin T, Twerenbold R, Mäder F, Zellweger C, Moehring B, Stallone F, Minners J, Rubini Gimenez M, Hoeller R, Murray K, Sou SM, Mueller M, Denhaerynck K, Mosimann T, Reiter M, Haaf P, Meller B, Freidank H, Osswald S, Mueller C. Serial changes in high-sensitivity cardiac troponin I in the early diagnosis of acute myocardial infarction. *Int J Cardiol* Elsevier Ireland Ltd; 2013;**168**:4103–4110.

6. Irfan A, Reichlin T, Twerenbold R, Meister M, Moehring B, Wildi K, Bassetti S, Zellweger C, Gimenez MR, Hoeller R, Murray K, Sou SM, Mueller M, Mosimann T, Reiter M, Haaf P, Ziller R, Freidank H, Osswald S, Mueller C. Early diagnosis of myocardial infarction using absolute and relative changes in cardiac troponin concentrations. *Am J Med* Elsevier Inc; 2013;**126**:781-788.e2.

7. Mueller M, Biener M, Vafaie M, Doerr S, Keller T, Blankenberg S, Katus H a, Giannitsis E. Absolute and relative kinetic changes of high-sensitivity cardiac troponin T in acute coronary syndrome and in patients with increased troponin in the absence of acute coronary syndrome. *Clin Chem* 2012;**58**:209–218.

8. Biener M, Mueller M, Vafaie M, Keller T, Blankenberg S, White HD, Katus HA, Giannitsis E. Comparison of a 3-hour versus a 6-hour sampling-protocol using high-sensitivity cardiac troponin T for rule-out and rule-in of non-STEMI in an unselected emergency department population. *Int J Cardiol* Elsevier Ireland Ltd; 2013;**167**:1134–1140.

9. Biener M, Giannitsis E, Lamerz J, Mueller-Hennessen M, Vafaie M, Katus HA. Prognostic value of elevated high-sensitivity cardiac troponin T levels in a low risk outpatient population with cardiovascular disease. *Eur Hear journal Acute Cardiovasc care* 2016;**5**:409–418.

10. Vasile VC, Saenger AK, Kroning JM, Jaffe AS. Biological and analytical variability of a novel high-sensitivity cardiac troponin T assay. *Clin Chem* 2010;**56**:1086–1090.

11. Wu AHB, Lu QA, Todd J, Moecks J, Wians F. Short- and long-term biological variation in cardiac troponin I measured with a high-sensitivity assay: implications for clinical practice. *Clin Chem* 2009;**55**:52–58.

12. Hammarsten O, Fu MLX, Sigurjonsdottir R, Petzold M, Said L, Landin-Wilhelmsen K, Widgren B, Larsson M, Johanson P. Troponin T percentiles from a random population sample, emergency room patients and patients with myocardial infarction. *Clin Chem* 2012;**58**:628–637.
